# Physics driven behavioural clustering of free-falling paper shapes

**DOI:** 10.1371/journal.pone.0217997

**Published:** 2019-06-26

**Authors:** Toby Howison, Josie Hughes, Fabio Giardina, Fumiya Iida

**Affiliations:** 1 Bio-Inspired Robotics Lab, Department of Engineering, University of Cambridge, Cambridge, United Kingdom; 2 School of Engineering and Applied Sciences, University of Harvard, Cambridge, United States of America; University of Vermont, UNITED STATES

## Abstract

Many complex physical systems exhibit a rich variety of discrete behavioural modes. Often, the system complexity limits the applicability of standard modelling tools. Hence, understanding the underlying physics of different behaviours and distinguishing between them is challenging. Although traditional machine learning techniques could predict and classify behaviour well, typically they do not provide any meaningful insight into the underlying physics of the system. In this paper we present a novel method for extracting physically meaningful clusters of discrete behaviour from limited experimental observations. This method obtains a set of physically plausible functions that both facilitate behavioural clustering and aid in system understanding. We demonstrate the approach on the V-shaped falling paper system, a new falling paper type system that exhibits four distinct behavioural modes depending on a few morphological parameters. Using just 49 experimental observations, the method discovered a set of candidate functions that distinguish behaviours with an error of 2.04%, while also aiding insight into the physical phenomena driving each behaviour.

## Introduction

Complex physical phenomena are often governed by highly non-linear, multidimensional dynamics. Hence, it can be challenging to understand these systems using traditional modelling tools, as we lack knowledge of the underlying physical phenomena required to implement these. The obvious course of action, then, is to infer these phenomena via physical experimentation. Automating this inference process, in other words automating the discovery of system physics from experimental data, has been the focus of intensive study.

Schmidt and Lipson [1] developed an algorithm to automatically discover analytical relationships in dynamical systems, ranging from simple harmonic oscillators to more complex chaotic double pendulum systems. This was preceded by a method of non-linear model synthesis from directly observed data using co-evolution [2]. Meanwhile, in the fluid dynamics community sparse regression has been used to determine the fewest terms in the dynamic governing equations required to accurately represent the data [3]. Data-driven approaches to modelling have also shown the ability to predict behaviours of dynamic systems [4, 5]. Similarly, big-data has been utilised for the prediction and physical understanding of complex systems include [6–10]. Other studies used evolutionary algorithms with feedback from environmental interaction to optimise robotic morphologies without any system model [11–13].

These approaches present a few problems. First is the reliance on large datasets. Sampling through physical experimentation typically involves searching high dimensional landscapes [14]. This makes data generation difficult, especially for expensive-to-evaluate functions. Second, although highly effective at identifying the inherent physical relationships of non-linear systems, they have not shown the ability to predict the boundaries of these non-linear behaviours. This is of particular importance in systems with a diverse range of discrete behavioural modes over their parameter space. In such systems, the dynamics of different behaviours may be significantly different, and the driving factors causing behavioural switches unclear. Such discrete behaviour systems are seen widely throughout nature including laminar-turbulent behaviours in fluid dynamics [15], gait patterns in locomotion [16, 17] or even the behaviour of flocking systems [18].

First proposed by Maxwell [19], one class of system that exhibits these characteristics is falling paper systems. For example, depending on properties such as diameter and fluid viscosity, circular disks of paper and other materials exhibit four distinct free-falling behaviours: steady falling, periodic oscillation, tumbling and chaotic falling [20]. There has been intensive study on modelling and understanding the behaviours of falling disks [21–27], rectangles [28–42] and other shapes such as parallelograms [43]. However, it is widely acknowledged that traditional modelling approaches such as solving the Navier–Stokes equations are intractable for this problem, with most approaches relying on assumptions such as one-dimensionality.

As a result, many studies focus on using experimental behavioural observations to understand the driving physical phenomena. Here, the approaches tend to characterise behaviours using dimensionless quantities such as the Reynolds number *Re* or dimensionless moment of inertia I* [30, 33, 36, 44, 45]. Using these quantities allows the construction of a dimensionless parameter space in which different regions correspond to different falling behaviours. Similar approaches using other dimensionless quantities such as the Froude *Fr* or Strouhal *St* numbers have been used in the analysis of behavioural diversity in other systems [26, 46–49]. The benefit of this method is that it facilitates a quantitative method to differentiate between behaviours, while also exposing the underlying physical phenomena in the system. However, it requires intensive testing of different dimensionless quantities to find those which are physically relevant to the system.

In this paper we present Physics Driven Behavioural Clustering (PDBC), a novel method that automates the process of discovering functions that enable behavioural clustering and physical understanding of systems with discrete behavioural modes. The PDBC method has the potential to discover physically insightful clustering functions based on relatively few experimental observations, thus enabling breakthroughs in the understanding of expensive-to-evaluate and behaviourally diverse systems.

In the PDBC method, observational data is organized and transformed into the parameter space of a set of generic functions. We hypothesize that there exists a set of functions whose parameter space is divided into distinct regions corresponding to different behavioural modes. Furthermore, we hypothesize that more physically relevant functions—such as *Re* and I* in the falling disk system—will cluster similar behaviours together more effectively. We propose that the predictive accuracy and clustering strength of a standard unsupervised clustering algorithm in this parameter space can be used as a direct metric for physical significance, with strongly clustered solutions with low predictive errors being more physically relevant.

We address the challenging problem of clustering and understanding the falling behaviours in the V-Shaped Falling Paper (VSFP) system, which is a new contribution to the falling paper system class. This is inspired by the falling and fluttering behaviours observed by helicopter seeds [50–52]. The VSFP system is an interesting challenge because although the design parameter space is limited, it exhibits rich behavioural diversity. Therefore it is an ideal system to demonstrate the PDBC method. We demonstrate the PDBC method can effectively cluster and help explain the VSFP behaviours.

This paper is structured as follows. First, we describe the PDBC method for a general system. Following this we describe the VSFP system. Next, we present experimental results of the VSFP system and the PDBC method. We discuss the effectiveness and physical significance of the results. Finally, we conclude and discuss further work.

## Materials and methods

### Physics-Driven Behavioural Clustering (PDBC)

The PDBC method is inspired by the idea of dynamic similarity, which uses dimensionless quantities to assess the similarity between different systems whose properties are not necessarily the same. For example, the flow of two fluids with different densities in pipes of differing diameters are said be similar if a dimensionless quantities—the Reynolds Number (Re)— is the same for each flow [53]. Furthermore, the value of Re indicates the flow behaviour, e.g. laminar or turbulent. Hence, dimensionless quantities can be used both for clustering and physical insight.

We hypothesise that for dynamic systems with discrete behavioural modes there exists a set of functions whose parameter space is divided into distinct regions– separated by transitional zones—corresponding to different behavioural modes. Although not strictly dimensionless, we expect these functions to represent the underlying structure of dimensionless quantities, and hence term them pseudo-dimensionless quantities (PDQ’s). We further hypothesise that the more effectively PDQ’s cluster similar behaviours together, the greater physical insight they contains.

**Fig 1:**
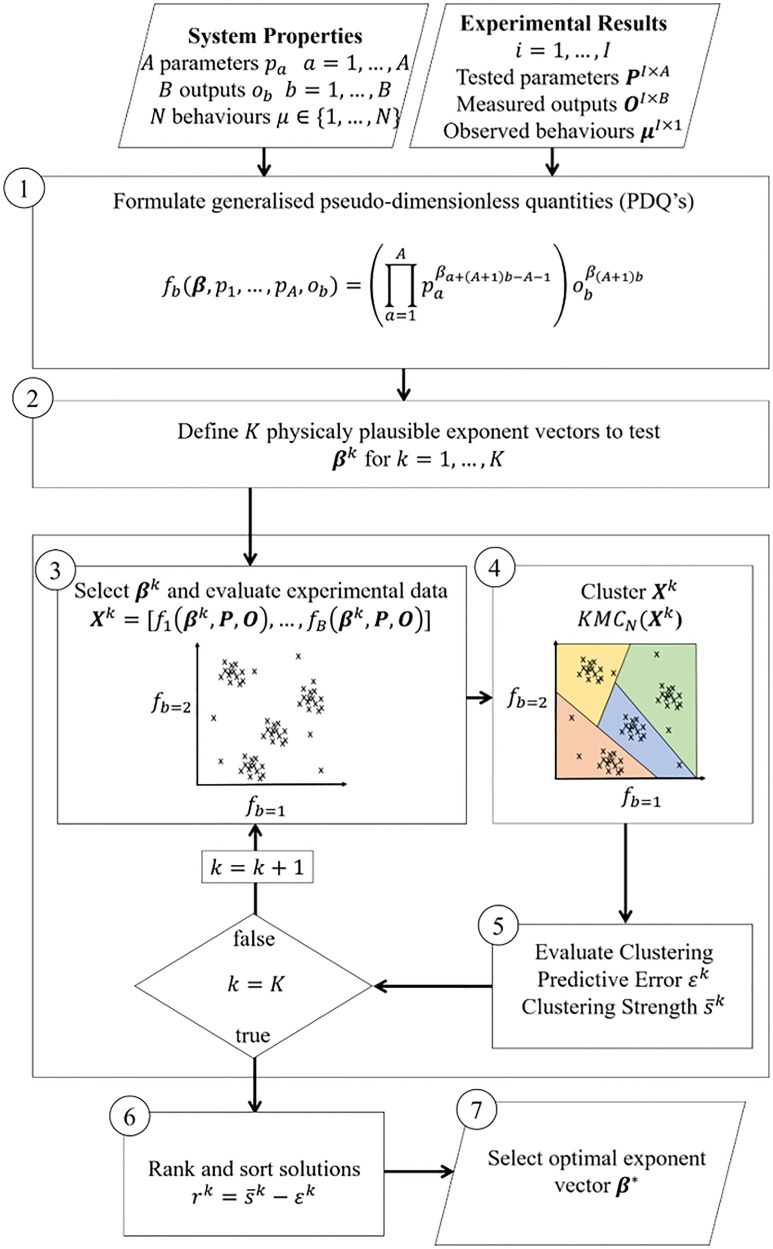
Flow chart of generalised physics driven behavioural clustering method. Experimental observations with different system parameters along with there corresponding behaviours are the input. (1) A set of general functions called PDQ’s are formulated for the system. (2) Using a heuristic physics based approach, we define a range of plausible values for the PDQ exponents. (3) We evaluate the PDQ’s with different combinations of exponents. (4-5) For each exponent combination we run a clustering algorithm in the PDQ parameter space and evaluate the predicted error of system behaviours, and the clustering strength. (6-7) We chose the exponent combination with the best trade off between minimizing predictive error and maximizing clustering strength.

PDBC is a formalised approach for searching through and evaluating different PDQ’s. Fig 1 shows a schematic of the process, which we explain individually in detail below.

#### Data acquisition and processing

The PDBC method is intended for use with systems that that exhibit discrete and distinctive behavioural patterns as certain system variables are changed. The input of PDBC is experimental data of such systems, containing a range of variables, behavioural patterns and outputs. These behavoural patterns should be distinguished during the data acqusision phase by the user, either visually or otherwise. Table 1 summarises each input of the process in detail.

**Table 1:** PDBC inputs.

Input	Symbol	Size	Description
System Parameters	*p*_*a*_	*a* = 1, …, *A*	System parameters that can be varied, leading to different behavoural modes.
System Outputs	*o*_*b*_	*b* = 1, …, *B*	System outputs than can be measured for all behavioural modes.
Behavioural Category	*μ*	*μ* ∈ {1, …, *N*}	Numeric identifiers for each of the *N* behavoural modes observed in the system.
Experimental System Parameters	$P=[\begin{array}{c} p_{1,\ldots ,A}^{1}\\ \vdots \\ p_{1,\ldots ,A}^{I} \end{array}]$	*I* × *A*	Array of system parameter combinations tested over *I* experiments.
Experimental System Outputs	$O=[\begin{array}{c} o_{1,\ldots ,B}^{1}\\ \vdots \\ o_{1,\ldots ,B}^{I} \end{array}]$	*I* × *B*	Array of system outputs measured over *I* experiments.
Experimental System Behaviours	$\mu =\left[\begin{array}{c} \mu^{1}\\ \vdots \\ \mu^{I} \end{array}\right]$	*I* × 1	Matrix of observed behavioural modes observed over *I* experiments

#### (1) Formulation of generic PDQ’s

The first step in the PDBC process is to forumlate a set of generic PDQ’s. As previously stated PDQ’s are representations of dimensionless quantities, so should describe some relationship between the system parameters and outputs. A review of many common dimensionless quantities shows this relationship is usually characterised by the product of system parameters and outputs, raised to some exponent. Hence, the generic PDQ’s should faciliate the testing of many different combinations of the system inputs, outputs and exponents.

To satisfy this requirement, we formulate generic PDQ’s as exponential equations including the system parameters, outputs and generic exponents *β*_*c*_ for *c* = 1, 2, …, *C*, with ***β*** = (*β*_1_, *β*_2_, …, *β*_*C*_) being the *exponent vector*. Each PDQ includes all system parameters but only one output, with each term having one exponent. This allows us to specifically explore the relationship between the system parameters and each output. The total number of generic exponents, then, is *C* = *AB* + *B* and the PDQ’s are formulated as follows

$$\begin{array}{c} f_{b}\left(\beta ,p_{1},\ldots ,p_{A},o_{b}\right)=(\prod_{a=1}^{A}p_{a}^{\beta_{a+(A+1)b-A-1}})o_{b}^{\beta_{a+(A+1)b-A}} \end{array}$$ (1)

where as described in Table 1 *a* = 1, …, *A* and *b* = 1, …, *B*. Using this formulation, we can generate any number, say *K*, of exponent combinations $\beta^{k}=\left(\beta_{1}^{k},\beta_{2}^{k},\ldots ,\beta_{C}^{k}\right)$ for *k* = 1, 2, …, *K*.

#### (2) Exponent search policy

Given the generic PDQ’s, we next define a policy to search through the possible exponent values previously described. The goal of this policy is to evaluate the physically plausible exponents for a particular parameter or output, while ignoring those which are physically unlikely. Hence, for each parameter and output the user should define an exponent range and discretization increment that give rise to a set of plausible exponents. We denote this algebraicly as follows. The *a*th parameter has an exponent range

$$\begin{array}{c} \beta_{a+(b-1)A+(b-1)}^{k}\in \left\{-\Pi_{a},-\Pi_{a}+\Delta_{a},\ldots ,\Pi_{a}-\Delta_{a},\Pi_{a}\right\} \end{array}$$ (2)

and the *b*th output has an exponent range

$$\begin{array}{c} \beta_{A+(b-1)A+(b-1)}^{k}\in \left\{-\Pi_{b},-\Pi_{b}+\Delta_{b},\ldots ,\Pi_{b}-\Delta_{b},\Pi_{b}\right\} \end{array}$$ (3)

where Π_*a*_ and Π_*b*_ define the minimum and maximum values for each parameter or output, and Δ_*a*_ and Δ_*b*_ the corresponding discretization increment. The total number of exponent combinations *K* is therefore

$$\begin{array}{c} K=\prod_{a=1}^{A}(\frac{2\Pi_{a}}{\Delta_{a}}+1)\prod_{b=1}^{B}(\frac{2\Pi_{b}}{\Delta_{b}}+1) \end{array}$$ (4)

Clearly, setting Π_*a*_, Π_*b*_, Δ_*a*_ and Δ_*b*_ requires a heuristic approach. For example, parameters with units of length may relate to inertial terms or their inverses, so Π = 4, and could be discretized with Δ = 0.5. Limiting the range or using a large increment may lead to useful PDQ’s being lost. However, increasing the range or using a low incremement vastly increases the compuational cost of the PDBC process.

The authors present Table 2 as a suggested guide for choosing reasonable exponent ranges for certain parameter and outputs types.

**Table 2:** Suggested exponent ranges and increments.

Term	Units	Π	Δ	Reasoning
Length	L	4	0.5	Includes inertial terms
Angle	-	4	0.5	Includes Inertial Terms
Linear Velocity	LT^−1^	2	0.5	Includes Energy Terms
Angular Velocity	T^−1^	2	0.5	Includes Energy Terms

#### (3-4) PDQ clustering

We seek to determine how, given an exponent vector ***β***^*k*^, the PDQ’s cluster similar behaviours together. To achieve this we apply the K-Means unsupervised clustering algorithm [54] on the PDQ parameter space. This partitions the experimental observations into *N* clusters—corresponding to the number of system behaviors—which can be evaluated for their predictive accuracy and clustering strength. As previously mentioned, we hypothesise that more physically meaningful PDQ’s will yield stronger and more accurate clustering.

We evaluate the experimental parameters and outputs **P** and **O** into the PDQ parameter space **X**^*k*^

$$X^{k}=[\begin{array}{ccc} f_{1}\left(\beta ,P_{1}^{1},\ldots ,P_{A}^{1},O_{1}^{1}\right) & \ldots & f_{B}\left(\beta ,P_{1}^{1},\ldots ,P_{A}^{1},O_{B}^{1}\right)\\ \vdots & \vdots & \vdots \\ f_{1}\left(\beta ,P_{1}^{I},\ldots ,P_{A}^{I},O_{1}^{I}\right) & \ldots & f_{B}\left(\beta ,P_{1}^{I},\ldots ,P_{A}^{I},O_{B}^{I}\right) \end{array}]$$ (5)

The K-Means clustering algorithm is applied to **X**, yielding

$$\begin{array}{c} v^{k}=KMC_{N}\left(X^{k}\right) \end{array}$$ (6)

where *N* is the number of clusters to form, in this case the number of system behaviours, and **v**^*k*^ is an *I* dimensional array of cluster assignments, with $v_{i}^{k}\in \left\{1,2,\ldots ,N\right\}$. As is standard practice, the algorithm is run multiple times, three in this case, to avoid clustering anomalies.

#### (5) Evaluation of clustering performance

We introduce two measures of clustering performance; predictive error *ϵ* and clustering strength $\bar{s}$.

*Predictive error*: K-Means is an unsupervised method, so the cluster assignments in **v**^*k*^ do not correspond to the behavioural labels in ***μ***. In order to associate clusters assignments with behavioural labels we define ${\hat{v}}_{k}$, in which we uniquely reassign cluster assignments such that the fraction of misclassified behaviours—the *predictive error* *ϵ*^*k*^—is minimized

$$\begin{array}{c} \left({\hat{v}}_{i}^{k}\neq v_{i}^{k}\right)\wedge \left(\exists !\;{\hat{v}}_{i}^{k}\in \left\{1,2,\ldots ,N\right\}\right)\Leftrightarrow \underset{{\hat{v}}^{k}}{\text{min}\;}\epsilon^{k} \end{array}$$ (7)

where

$$\begin{array}{c} \epsilon^{k}=\frac{1}{I}\sum_{i=1}^{I}e_{i}^{k} \end{array}$$ (8)

and

$$\begin{array}{c} e_{i}^{k}\{\begin{array}{cc} 1 & {\hat{v}}_{i}^{k}\neq \mu_{i}\\ 0 & {\hat{v}}_{i}^{k}=\mu_{i} \end{array} \end{array}$$ (9)

*Clustering Strength*: We use the silhouette criterion [55] $s_{i}^{k}\in \left[-1,1\right]$ to quantify the clustering strength. $s_{i}^{k}$ is a measure of data consistency within clusters, representing how similar the *i*th observation is to its own cluster, relative to other clusters. The higher *s*, the stronger the clustering assignment for a particular observation is. We define the clustering strength as the mean of $s_{i}^{k}$ for all observations, e.g.

$$\begin{array}{c} {\bar{s}}^{k}=\frac{1}{I}\sum_{i=1}^{I}s_{i}^{k} \end{array}$$ (10)

#### (6-7) Optimal exponent vector selection

At this point in the PDBC process, all candidate exponent vectors have been evaluated for their predictive error *ϵ*^*k*^ and clusterring strength ${\bar{s}}^{k}$. Hence, we must define a measure of optimality by which we sort the PDQ’s from the most to the least physically insightful. We denote the optimal PDQ exponent vector as $\beta^{k^{*}}$.

Solutions with a low predictive error are desirable, as under our hypothesis these PDQ’s are likely to be more physically insightful. However, if the exponent search space is large there may be multiple solutions with a low predictive error; some arising from physically significance and some arising coincidentally. Hence, we must also consider the clusterring strength of the solution, with stronger clusterring also indicating more physical insight.

To achieve this we introduce the exponent ranking factor *r*^*k*^, that rewards strongly classified solutions with low predictive error. It is simple the sum of −*ϵ*^*k*^ and ${\bar{s}}^{k}$.

$$\begin{array}{c} r^{k}={\bar{s}}^{k}-\epsilon^{k} \end{array}$$ (11)

Hence, the optimal exponent vector $\beta^{k^{*}}$ corresponds to the highest rank $r^{k^{*}}$, and represents the PDQ’s with the best trade-off between predictive error and clustering strength. To ease the interpretation of the rankings we can sort the solutions in descending order in terms of *r*^*k*^, defining the solution rank number $\hat{k}\left(r^{k}\right)$ such that

$$\begin{array}{c} r^{\hat{k}\left(r^{k}\right)-1}\leq r^{\hat{k}\left(r^{k}\right)}\leq r^{\hat{k}\left(r^{k}\right)+1} \end{array}$$ (12)

for $\hat{k}\left(r\right)\in \left\{1,\ldots ,K\right\}$. Hence, the optimal solution $r^{k^{*}}$ corresponds to $\hat{k}\left(r^{k^{*}}\right)=1$, with solutions $\hat{k}\left(r^{k}\right)=2,\ldots ,K$ decreasing in their optimality.

### V-shaped falling paper system

To test the PDBC method we created the V-Shaped Falling Paper (VSFP) System, in which the passive falling behaviours of a V-shaped paper structure with an affixed mass are studied. The VSFP is a novel addition to the falling paper class of systems, and is to our knowledge unstudied. Here, we describe the VSFP system and experimental procedure, in the context of the PDBC method.

#### System morphology

**Fig 2:**
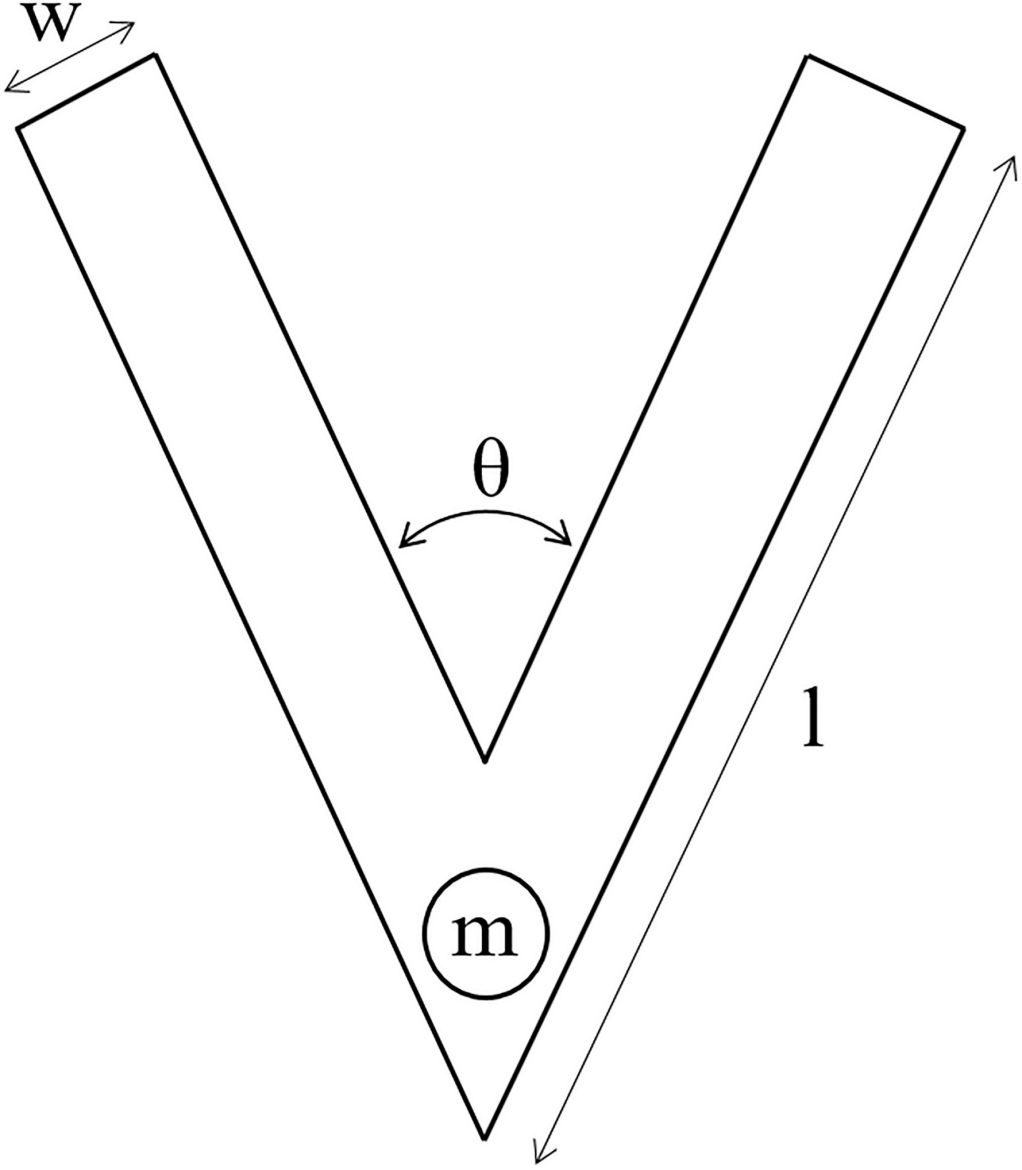
A parametrized paper V-shape with an affixed mass. The variable parameters are wing length *l* and wing angle *θ*. The fixed parameters are wing width *w* = 10mm and affixed mass *m* = 5g.

We study the passive falling behaviours of a V-Shaped piece of paper with an affixed mass; together termed a *structure*. The morphology of a structure is fully defined by the four parameters shown in Fig 2: the wing length *l*, wing angle *θ*, wing width *w* and affixed mass *m*. *l* and *θ* may vary, while *w* and *m* are fixed at 10mm and 5g. Hence, the two system parameters to be used in the PDBC method are *p*_1_ = *l* and *p*_2_ = *θ*.

#### System behaviours

**Fig 3:**
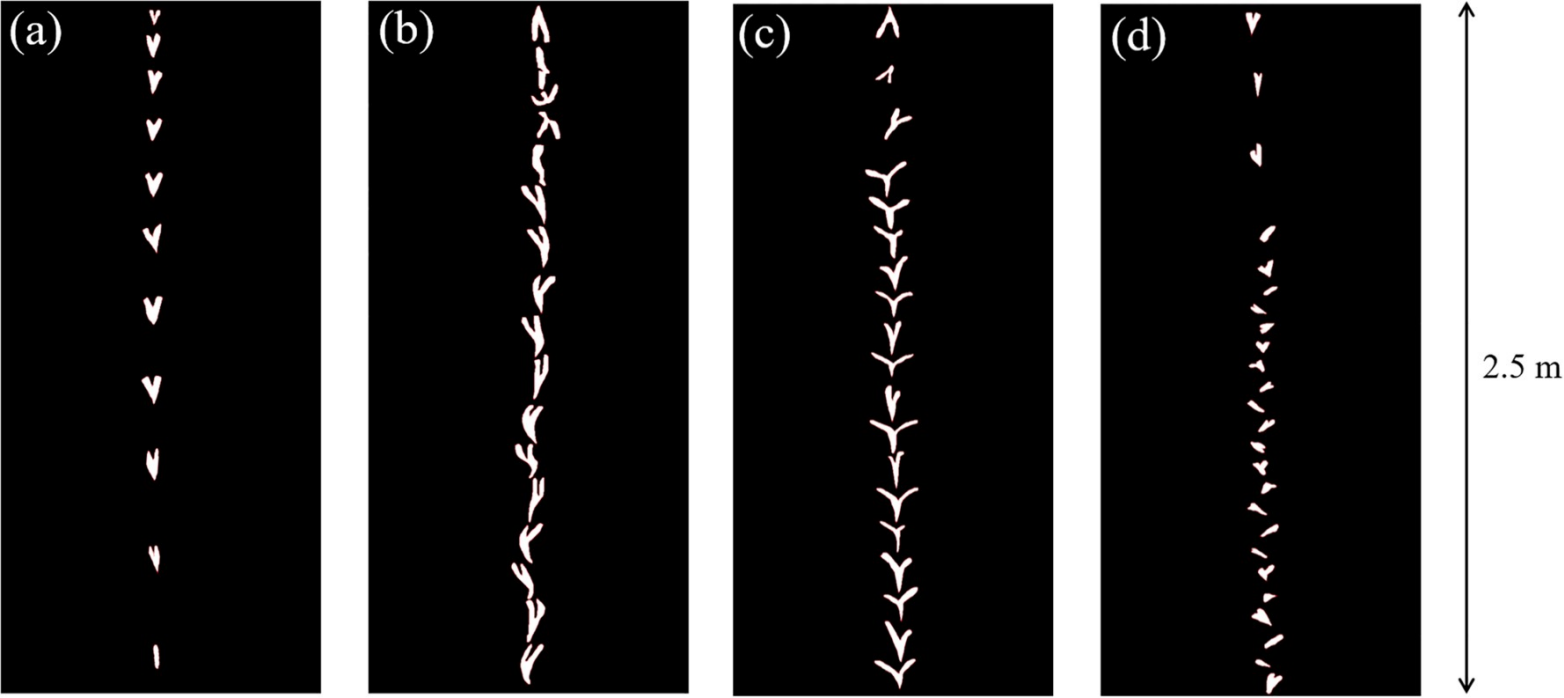
Snapshot images of the four directly observable behaviours in the VSFP system. (a) Plummeting (b) Undulating (c) Helicopter rotation (d) Asymmetric rotation. These snapshots show the structures falling a height of 2.5m.

As the morphological parameters *l* and *θ* are varied, the passive falling behaviours change. During free-fall, structures exhibit a transient and steady state phase; when falling they may rapidly pass through more than one behaviour before settling on a single behaviour. In this study, we neglect the transient phase as we found it to be highly unpredictable. Hence, the output of each drop test is the steady state behaviour. Four behavioural modes are directly observable; plummeting, undulating, helicopter rotation and asymmetric rotation. Fig 3 shows example snapshots of each of these while Table 3 outlines the characteristics of each behaviour; see also S1 Video. The rotative behaviours (c,d) are easily distinguishable from each other and the non-rotative behaviours (a,b).

**Table 3:** Qualitative description of behavioural modes of paper shapes.

Behavioural Mode	Characteristics
Plummeting	Shape falls directly to the ground with no wing movement.
Undulating	Shape falls directly to the ground, wings oscillate.
Helicopter Rotation	Wings splay in either direction, shape rotates to the ground.
Asymmetric Rotation	Shape rotates around mass.

#### System outputs

**Fig 4:**
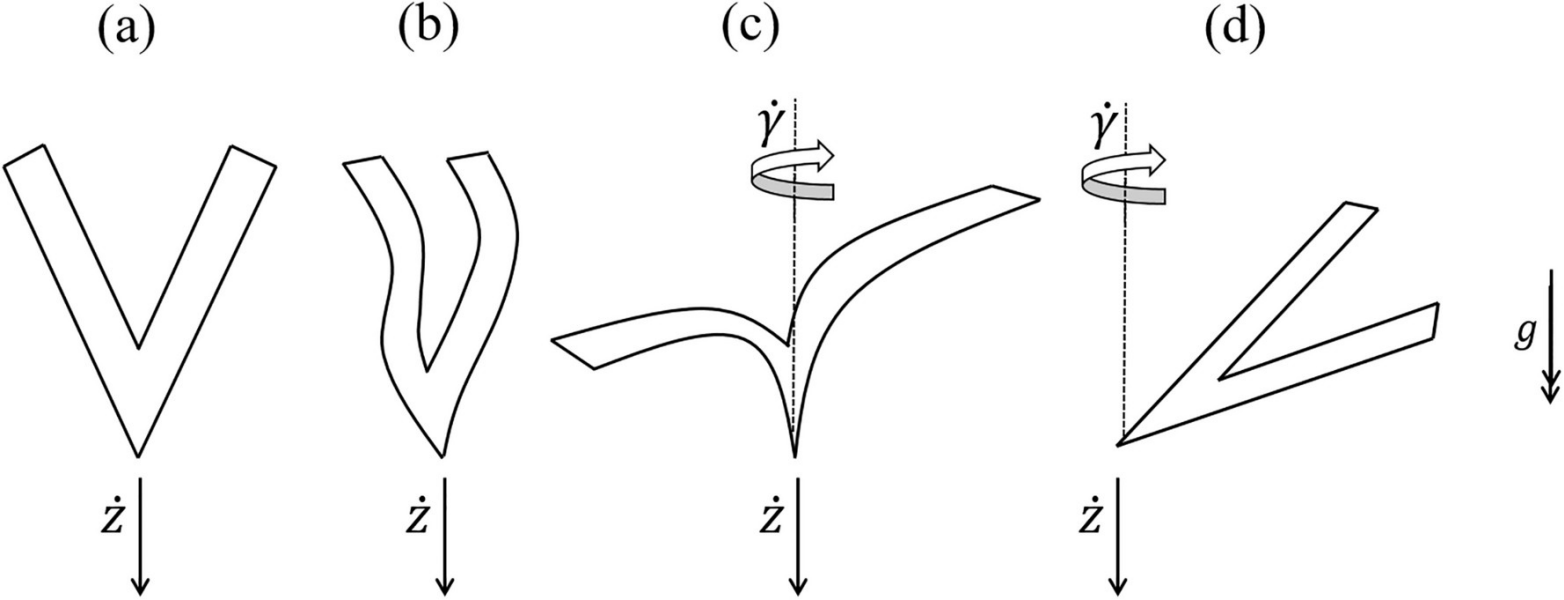
Diagrammatic representation of the four different behaviours, showing the measured outputs $\dot{z}$ and $\dot{\gamma }$. For the non-rotative behaviours (a,b) $\dot{\gamma }=0$.

There are many possible outputs, such as falling speed, rotation speed, rotation angle, oscillatory frequency or horizontal speed; some of these are only measurable in certain behavioural modes. The PDBC method is designed to be used with *universally measurable outputs*, which we define as being observable in all behavioural modes. In the case of the VSFP system, this limits the outputs to falling speed $\dot{z}$ and rotation speed; the rotational speed of the plummeting and undulating behavioural modes being negligible, but measurable. $\dot{\gamma }$. Fig 4 shows a schematic of the different behaviours and universally measurable outputs.

### Experimental procedure

#### Manufacturing

**Fig 5:**
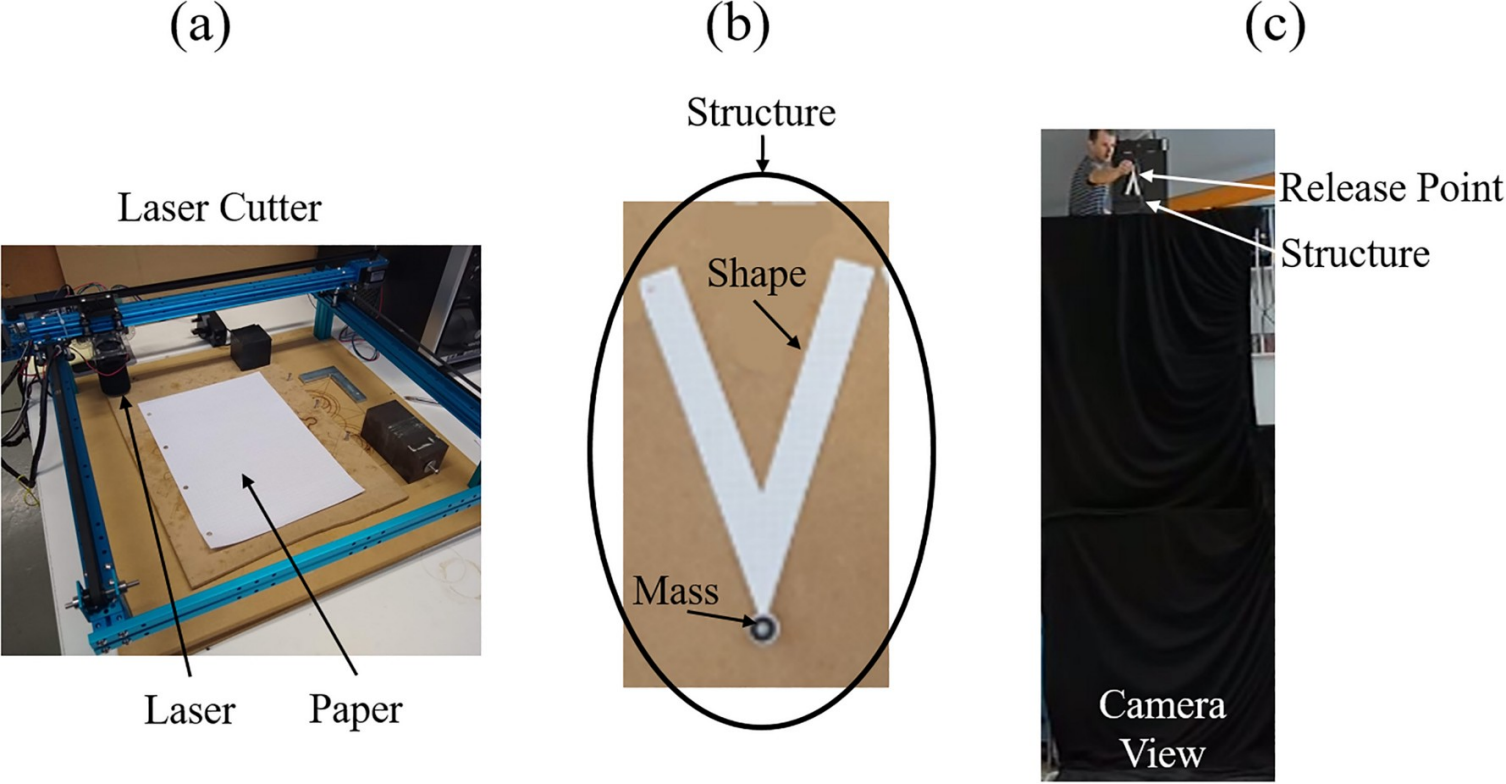
The experimental set-up, showing (a) Endurance MakeBlock XY engraving/cutting machine (b) structure comprising of paper shape and affixed mass (c) camera view of experimental drop zone.

**Fig 6:**
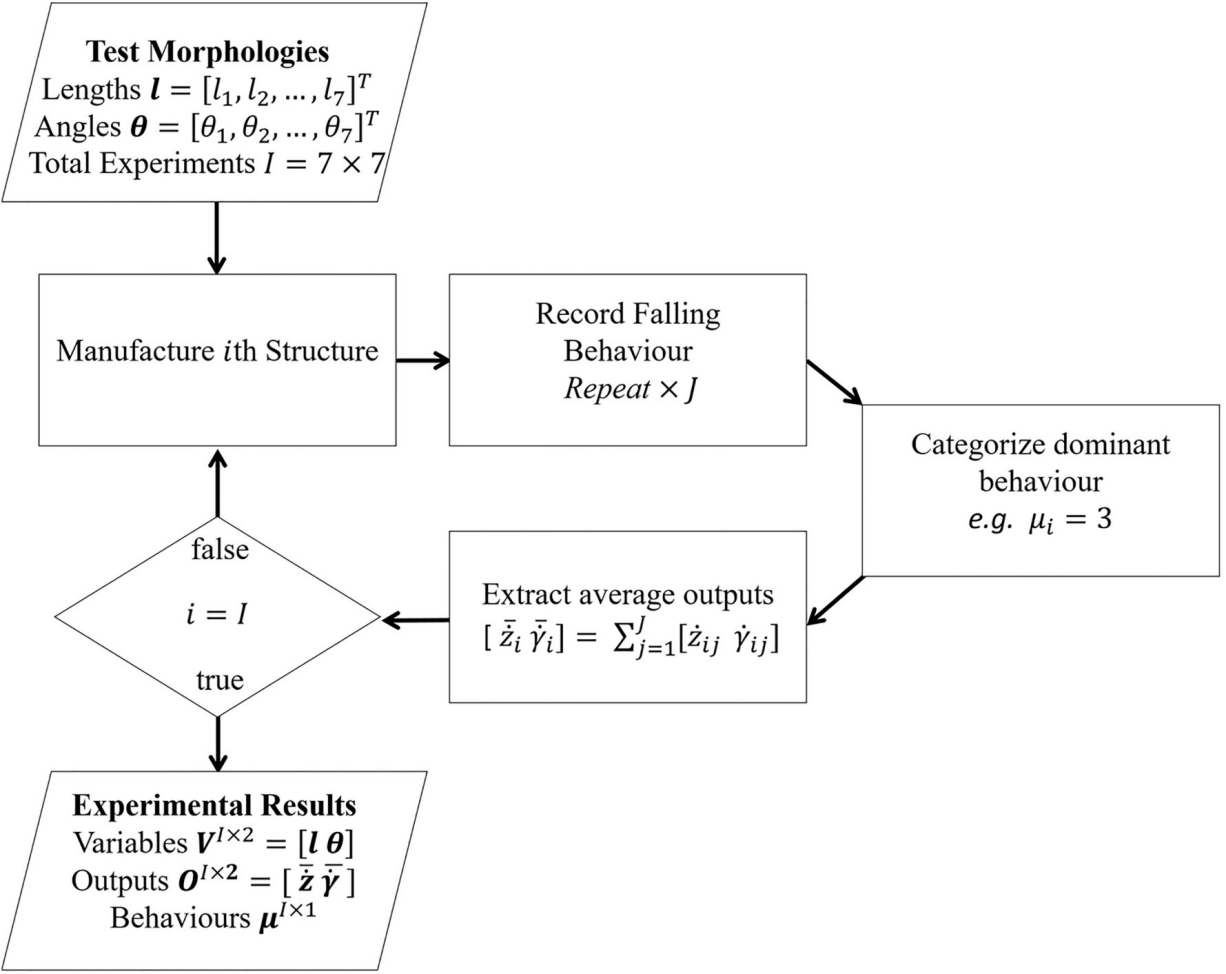
Flow chart of the experimental procedure. Structures are manufactured using the laser cutter. Structures are dropped and recorded *J* = 5 times. The dominant falling behaviour is extracted across these trials. The measured outputs are extracted and average over these trials, yielding $\bar{\dot{z}}$ and $\dot{\bar{\gamma }}$. The process is repeated for every combination of *l* and *θ* in the search space.

An Endurance MakeBlock XY engraving/cutting machine—as shown in Fig 5a—was to cut the shape out of Silvine A4 Graph Refill paper. The paper has a weight of 80 grams per square metre. The mass—for which 2 standard M4 steel washers were used— was affixed to the tip using superglue, with one washer on either side of the shape. Fig 6 shows the experimental procedure.

#### Testing

Each structure—as shown in Fig 5b—was manually dropped from a height of 3m into still air and using a tip up initial condition, as shown in Fig 5c. Structures fell against a black backdrop, and were recorded using a Logitech BRIO camera recording at 120 fps. The system outputs $\dot{\gamma }$ and $\dot{z}$ were manually extracted from the video data. Each structure was dropped *J* = 5 times, and the average outputs $\bar{\dot{\gamma }}$ and $\bar{\dot{z}}$ were calculated

$$\begin{array}{c} \left[{\bar{\dot{\gamma }}}_{i}\;{\bar{\dot{z}}}_{i}\right]=\frac{1}{J}\sum_{j=1}^{J}\left[{\dot{\gamma }}_{i}^{j}\;{\dot{z}}_{i}^{j}\right] \end{array}$$ (13)

## Results

### VSFP experimental results

A series of structures were manufactured and their behaviours recorded, as previously described. The PDBC method was applied to these results with the aim of clustering the system behaviours and gaining physical insight into the system. In this section we describe the VSFP results, including the type of behaviours observed, their outputs and any relationship to *l* and *θ*.

The *l* − *θ* parameter search space was discretized such that

$$\begin{array}{c} l\in \{75,95,115,135,155,175,195\}\;(\text{mm}) \end{array}$$ (14a)

$$\begin{array}{c} \theta \in \{30,37.5,45,52.5,60,67.5,75\}\;(\text{deg}) \end{array}$$ (14b)

**Fig 7:**
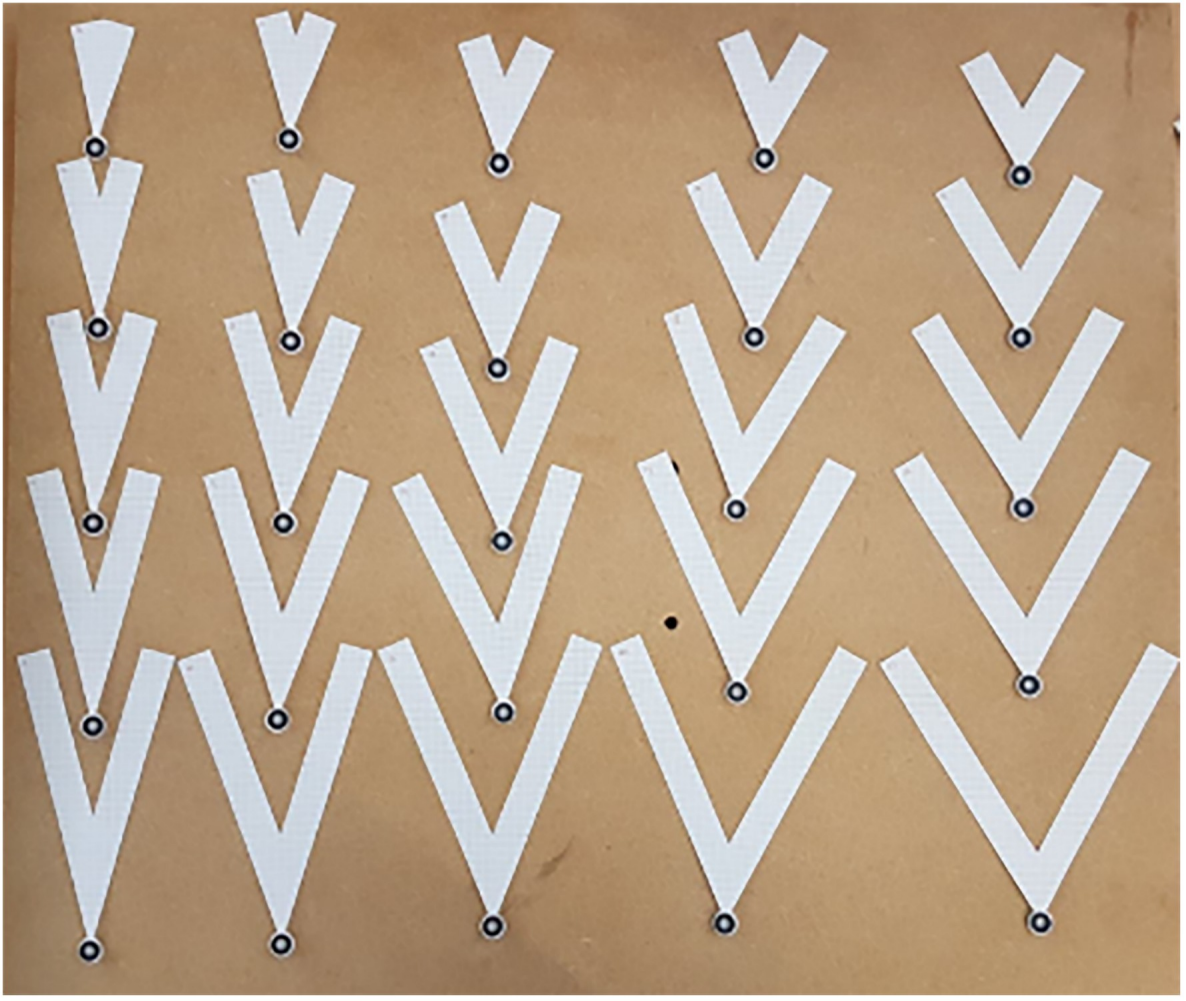
Example of manufactured and tested structures.

Hence, a total of *I* = 49 structures were tested, some of which are shown in Fig 7. First, we describe the results of these experiments.

#### Behavioural diversity and structure

**Fig 8:**
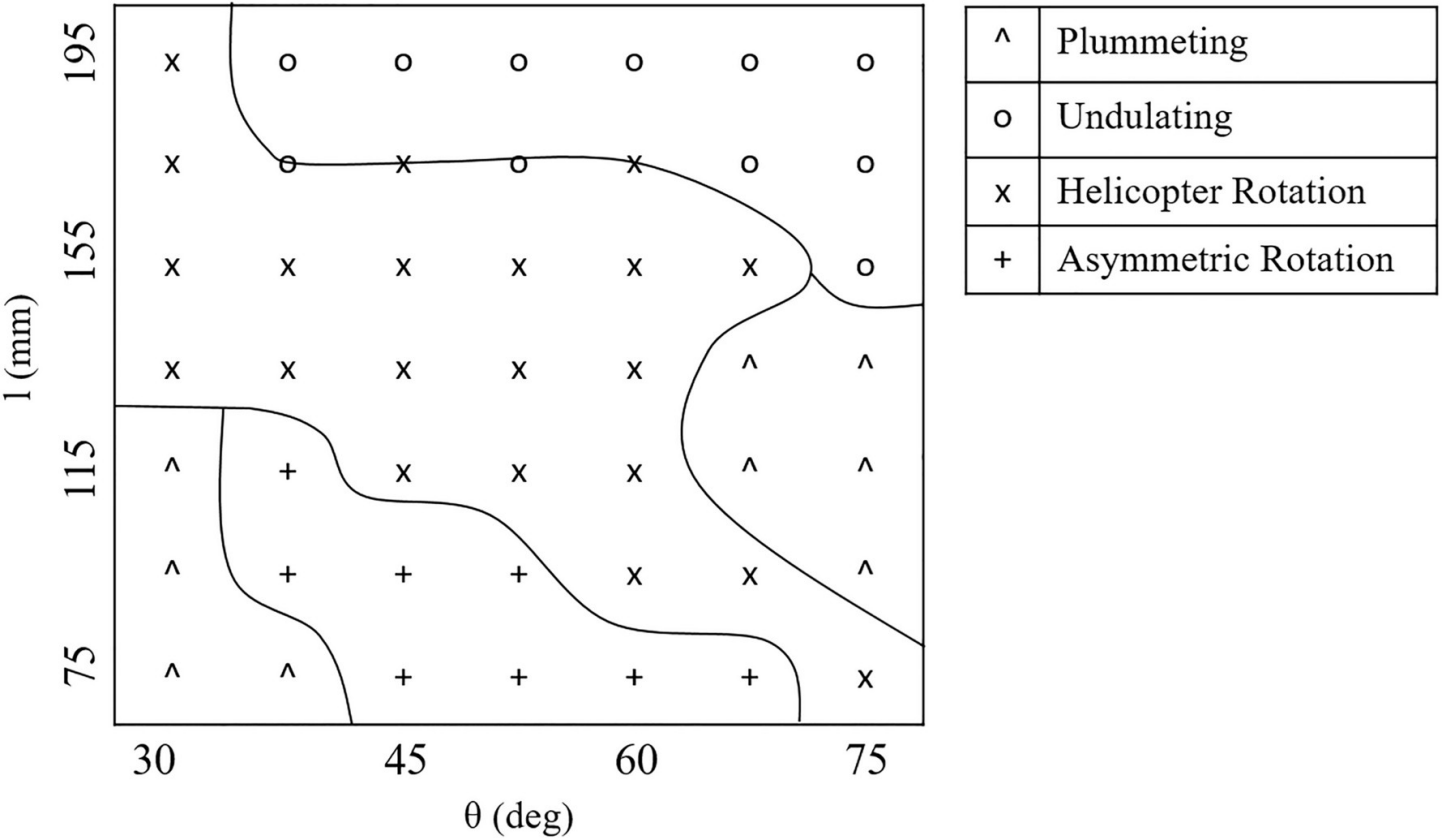
Dominant system behaviours across the morphological search space. Each behaviour is marked with a symbol, as shown in the legend. Lines have been added to estimate where the boundary between behaviours lies.

There are four observable behavioural modes, as described in Table 3. Fig 8 shows the observed dominant behaviour as a function of *l* and *θ*, which were also stored in the behavioural ground-truth vector ***μ***. There are five distinct behavioural regions; lines have been added by hand to indicate their approximate boundaries. Despite this apparent structure, there is no obvious rule to differentiate between behaviours based solely on *l* and *θ*. Morphologies with *l* ≥ 155mm are dominated by undulating behaviour across all angles except 30°. These morphologies have long wings with a range of angles. Morphologies with *l* ≤ 95mm are dominated by asymmetric rotation, except at the limits of *θ* ≤ 37.5^*o*^ and *θ* = 75^*o*^. These morphologies have short wings with a smaller range of angles. Plummeting behaviours can be observed in morphologies with *l* ≤ 115mm and *θ* ≤ 37.5^*o*^, and also morphologies with 95mm ≤ *l* ≤ 135mm and *θ* ≥ 67.5^*o*^. Plummeting is the only behaviour observed in two distinct regions of the morphological search space, with the morphologies having either short wings with a low angle or mid-length wings with a high angle. The helicopter rotation region spans a range of *l* and *θ*. At the lower boundary *l* increases as *θ* decreases. The upper boundary is less well defined, with a general transition from to plummeting behaviours.

#### Behavioural outputs

**Fig 9:**
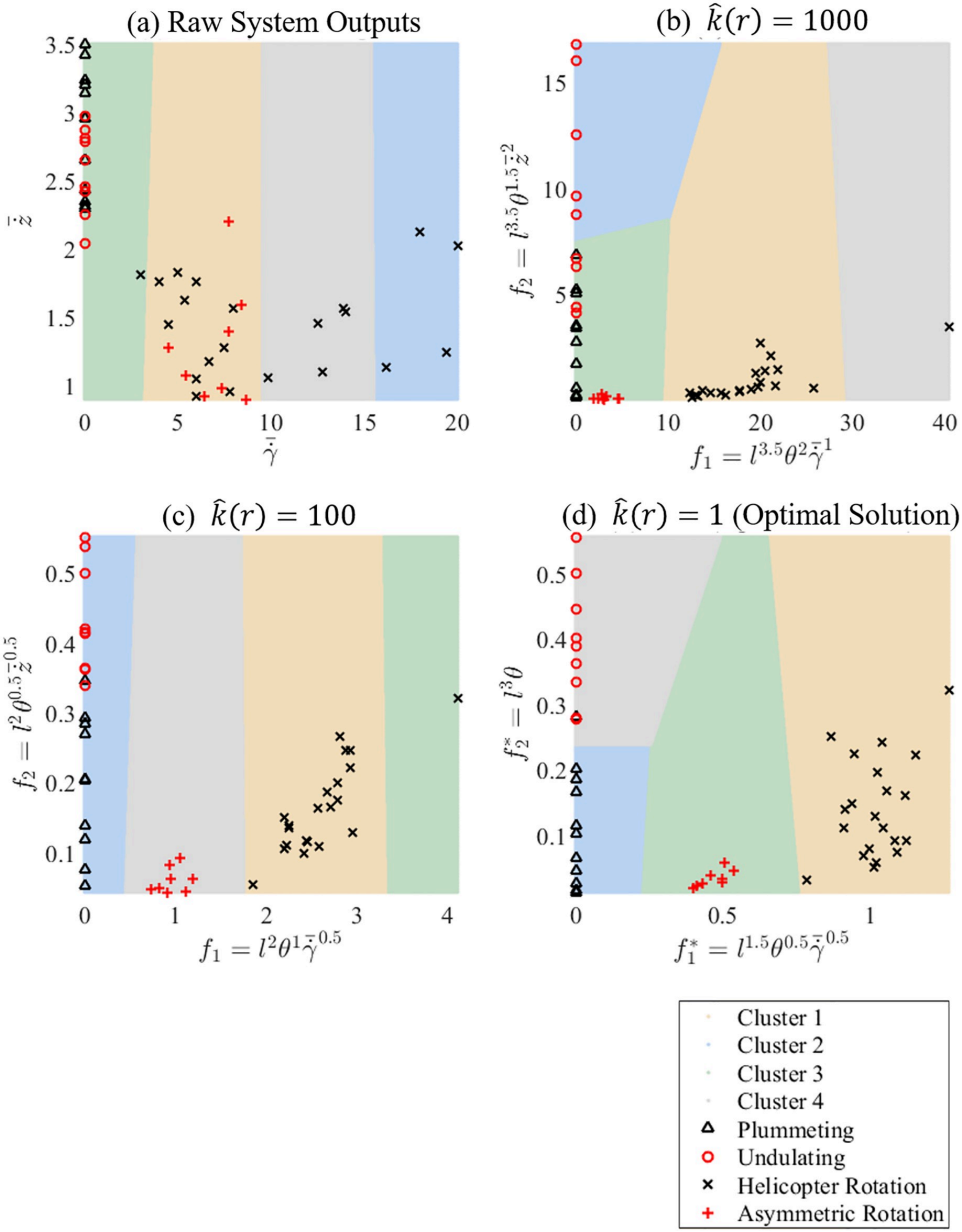
Clustering solutions for (a) raw system outputs, (b) 1000th ranked, (c) 100th ranked and (d) optimal exponent vector.

The behavioural outputs $\bar{\dot{z}}$ and $\bar{\dot{\gamma }}$ were extracted. Fig 9a shows these outputs plotted against each other, as well a clustering regions which demonstrate the need for the PDBC method. The full results set can be found in S1 Table. There is a clear distinction between the rotating and non-rotating behaviours. The plummeting and undulating observations are non-rotating, i.e. $\bar{\dot{\gamma }}=0$ so the output space is one-dimensional. The helicopter and asymmetric rotation behaviours have non-zero $\bar{\dot{\gamma }}$ and $\bar{\dot{z}}$ components. Plummeting behaviours range from 2.4m/s to 3.5 m/s in $\bar{\dot{z}}$ and 0rad/s in $\bar{\dot{\gamma }}$. Undulating behaviours range from 2.1m/s to 3.9 m/s in $\bar{\dot{z}}$ and 0rad/s in $\bar{\dot{\gamma }}$. Helicopter rotation behaviours range from 0.9m/s to 2.1 m/s in $\bar{\dot{z}}$ and 3rad/s to 20rad/s in $\bar{\dot{\gamma }}$. Asymmetric rotation behaviours range from 0.9m/s to 2.1 m/s in $\bar{\dot{z}}$ and 5rad/s to 9rad/s in $\bar{\dot{\gamma }}$.

In general, non-rotative behaviours almost exclusively fall faster than rotative behaviours. Within this, plummeting behaviours tend to fall faster than undulating behaviours. For rotative behaviours there is no clear behaviour that falls fastest or slowest. Nor is there an obvious relationship between falling speed and rotation speed, with the range in $\bar{\dot{z}}$ being similar for both behaviours. However, the $\bar{\dot{\gamma }}$ in helicopter rotation is around four times that of asymmetric rotation.

### PDBC results

We applied to PDBC method to the VSFP system with the aim of discovering a set of functions to classify the *N* = 4 observed behaviours and infer physical significance from this clustering. In the VSFP system there are two variables *l* and *θ*, and two outputs $\bar{\dot{z}}$ and $\bar{\dot{\gamma }}$. Hence, *A* = 2 and *B* = 2 so we formulate two PDQ’s with a total of *C* = 6 exponent, yielding

$$\begin{array}{c} f_{1}\left(\beta^{k},l,\theta ,\bar{\dot{z}}\right)=l^{\beta_{1}^{k}}\theta^{\beta_{2}^{k}}{\bar{\dot{\gamma }}}^{\beta_{3}^{k}} \end{array}$$ (15a)

$$\begin{array}{c} f_{2}\left(\beta^{k},l,\theta ,\bar{\dot{\gamma }}\right)=l^{\beta_{4}^{k}}\theta^{\beta_{5}^{k}}{\bar{\dot{z}}}^{\beta_{6}^{k}} \end{array}$$ (15b)

where the exponent vector ranges were set following those described in Table 2

$$\begin{array}{c} \beta_{1,2,4,5}^{k}\in \left\{-4,-3.5,-3,-2.5,-2,-1.5,-1,-0.5,0,0.5,1,1.5,2,2.5,3,3.5,4\right\} \end{array}$$ (16a)

$$\begin{array}{c} \beta_{3,6}^{k}\in \left\{-2,-1.5,-1,-0.5,0,0.5,1,1.5,2\right\} \end{array}$$ (16b)

Hence, the total number of exponent vectors to test was *K* = 6765201.

#### Optimal exponent vector

After examining all exponent vectors, we ranked the solutions with respect to the criterion specified by (11). While most of these solutions do not cluster the experimental data well as shown in Fig 9b and 9(c), the highly ranked solutions show a clear structures in the output space, the best of which is shown in Fig 9d. The highest ranked solution has a predictive error *ϵ* = 0.0204—corresponding to one misclassified behaviour—and a clustering strength $\bar{s}=0.8581$. The optimal exponent vector was

$$\begin{array}{c} \beta^{*}=\left(1.5,0.5,0.5,3,1,0\right) \end{array}$$ (17)

which corresponds to optimal PDQ’s of the form

$$\begin{array}{c} f_{1}^{*}=l^{1.5}\theta^{0.5}{\bar{\dot{\gamma }}}^{0.5} \end{array}$$ (18a)

$$\begin{array}{c} f_{2}^{*}=l^{3}\theta \end{array}$$ (18b)

The plummeting and undulating behaviours both have $f_{1}^{*}=0$. Hence, they can be distinguished using only $f_{2}^{*}$, with the region $f_{2}^{*}<0.24$ being characterised by plummeting behaviours and $f_{1}^{*}>0.24$ characterised by undulating behaviours. The single misclassified behaviour is a plummeting behaviour that has been incorrectly clustered with the undulating behaviours at $f_{2}^{*}=0.275$. The asymmetric rotation behaviours are tightly clustered together with $0.4<f_{1}^{*}<0.575$ and $f_{2}^{*}<0.075$. The helicopter rotation behaviours are more scattered, with $0.79<f_{1}^{*}<1.25$ and $f_{2}^{*}<0.35$. $f_{1}^{*}$ can be used to distinguish between the helicopter rotation, asymmetric rotation and plummeting/undulating behaviours combined. Only when combined with $f_{2}^{*}$ can all four behaviours be distinguished in the PDQ parameter space.

#### Solution landscape

**Fig 10:**
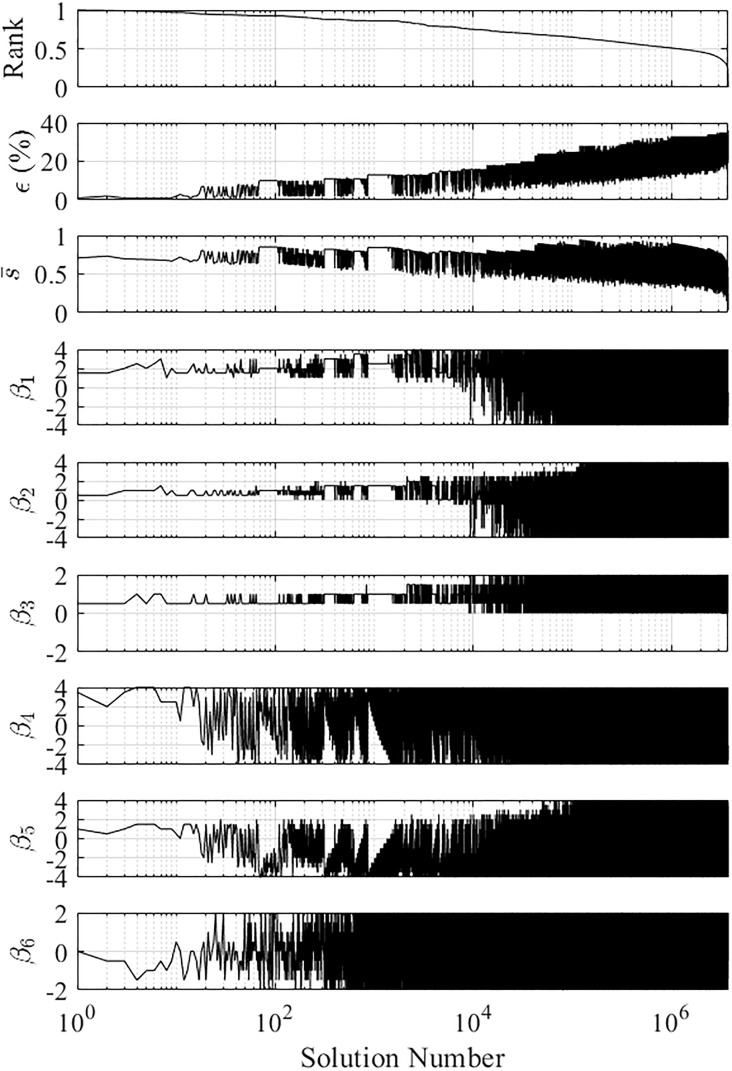
Solution landscape for all tested exponent vectors, showing the rank *r*^*k*^, predictive error *ϵ*, clustering strength ${\bar{s}}^{k}$ and exponent values *β*_1,…,6_.

As well as the optimal solution, we examined the landscape of all *K* exponent vectors. Fig 10 shows the *r*^*k*^, *ϵ*^*k*^, ${\bar{s}}^{k}$ and *β*_1,…,6_ for all tested solutions, sorted in decreasing rank order. The main characteristics of the solution landscape a presented here.

*r*^*k*^, *ϵ*^*k*^ *and* ${\bar{s}}^{k}$: The top-ten highest ranked solutions all have *ϵ*^*k*^ ≤ 0.0612, with six solutions having the minimum *ϵ*^*k*^ = 0.0204. Correspondingly, for these ten solutions ${\bar{s}}^{k}\approx 0.75$. Beyond this, the predictive error increases to a maximum of *ϵ*^*k*^ = 0.5510, while the clustering strength decreases to a minimum of ${\bar{s}}^{k}=0.2959$. Across this trend, there are many solutions with a high ${\bar{s}}^{k}$, indicating strong clustering. However, they correspond to low *ϵ* values, so are not ranked highly. This shows that strong clustering can be achieved regardless of *ϵ*, reinforcing the need to consider both *ϵ* and ${\bar{s}}^{k}$. As *ϵ* increases and $\bar{s}$ decreases, the *r*^*k*^ decreases to zero, as these solutions are neither distinguish between behaviours or exhibit strong clustering.

*β*_1_, *β*_2_ *and β*_3_: These are the exponents corresponding to the first PDQ (15a). Over the top 1000 solutions, 55% of *β*_1_, 52% of *β*_2_ and 98% of *β*_3_ values remain with ±0.5 of the optimum values of 1.5, 0.5 and 0.5 respectively. After this point, they begin to vary more. *β*_2_ is limited to the range [0 2], since negative values were unable to computed as they resulted in a division by zero.

*β*_4_, *β*_5_ *and β*_6_: These are the exponents corresponding to the second PDQ (15b). Over the top 1000 solutions, 5% of *β*_4_, 7% of *β*_5_ and 5% of *β*_6_ values remain with ±0.5 of the optimum values of 1.5, 0.5 and 0.5 respectively.

Fig 9b and 9c shows representative clustering solutions for the 100th and 1000th highest ranked exponent vectors. We can see that as the solution rank increases, the grouping of behavioural groups increases, while the separation between groups decreases. The exponent vector ***β***^*k*^ = (0, 0, 1, 0, 0, 1) corresponds to PDQ’s using just the raw measured outputs $\bar{\ldots \gamma }$ and $\bar{\dot{z}}$. This is an interesting solution to examine as the PDBC method is predicated on the notion that the raw outputs alone are not enough to distinguish between system behaviours. This was confirmed, as the solution had a ranking number of $\hat{k}\left(r\right)=35568$ with *ϵ*^*k*^ = 0.5102 and ${\bar{s}}^{k}=0.8550$. Fig 9a shows this clustering solution.

#### Physical significance

Inferring physical significance from the PDBC results is challenging, but some general statements can be made. We consider the optimal PDQ’s $f_{1}^{*}$ and $f_{2}^{*}$ separately.

$f_{1}^{*}=l^{1.5}\theta^{0.5}{\bar{\dot{\gamma }}}^{0.5}$: The analysis of the solution landscape showed that the performance ranking was highly sensitive to exponent changes in this PDQ. This is particularly the case for $\bar{\dot{\gamma }}$, which is strongly dependant on an exponent of 0.5. Hence, we can infer that this term is key in understanding each behaviour. Furthermore, we can show that $f_{1}^{*}$ may represent some form of the Reynolds number *Re*. First, we observe that the term $l\dot{\gamma }$ can represent the wing tip velocity of the rotative structures. Defining this as $V_{\text{tip}}=l\dot{\gamma }$, we can recast $f_{1}^{*}$ as

$$\begin{array}{c} f_{1}^{*}={\left(l\theta \right)}^{0.5}{\left(lV_{\text{tip}}\right)}^{0.5} \end{array}$$ (19)

The Reynolds number is the ratio of a velocity and length term to the kinematic viscosity *ν* of the liquid under study. In the VSFP system *ν* is the kinematic viscosity of the air in which the structures fall, and remains unchanged between all experimental observations. Hence, *lV*_tip_ = *νRe*, and we can recast (19) as

$$\begin{array}{c} f_{1}^{*}={\left(l\theta \right)}^{0.5}{\left(\nu Re\right)}^{0.5} \end{array}$$ (20)

Hence, following the physical meaning of *Re*, the rotative behaviours may be characterised by the ratio of inertial to viscous forces at their wing tip, relative to *lθ*, which is a shape parameter representing the wing length and angle. For the non-rotative behaviours, this analysis doesn’t apply as the rotation speed is zero.

$f_{2}^{*}=l^{3}\theta$: The analysis of the solution landscape showed that the performance ranking is less sensitive to exponent changes in this PDQ. Interestingly, the optimal PDQ doesn’t rely on $\bar{\dot{z}}$ at all. The terms in the PDQ are harder to interpret, but they tell us that the transition between the plummeting and undulating behaviours is governed by the wing length cubed multiplied the wing angle. This term is very similar to a moment of inertia term, indicating that this transition is related to the ease with which the structure can rotate or oscillate relative to the airflow.

## Discussion

In this paper we presented the PDBC method as a framework for clustering and aiding understanding of systems with discrete behavioural modes. Furthermore, we presented the VSFP problem, a new category of falling paper systems, and applied the PDBC method to it.

The PDBC method is the main contribution of this paper. The results indicate that the PDBC method is an effective way of finding a parameter space in which behaviours can be clustered together with a high degree of accuracy, with the optimal exponent vector having a predictive error of just *ϵ* = 0.0204. In terms of physical significance, the optimal PDQ’s showed that behaviours can be clustered and categorized using a variant of the rotative Reynolds number on one axis, and a shape factor similar to the moment of inertia on the other. Interestingly, this is a relationship that is common among falling paper problems with [20, 30], for example, reporting a similar behavioural relationship. Hence, this reinforces the hypothesis that choosing the most accurate and strongest clustered solutions reveals physically significant PDQ’s.

The VSFP system represents another significant contribution of this paper. The majority of falling paper systems consider rigid, or almost-rigid, objects as this eases the aerodynamic analysis. The VSFP system departs from this, with two of the four system behaviours—helicopter rotation and undulating—relying on large amounts of deformation. Allowing for such deformation yields a system with rich, varying and beautiful behaviours. However, these behaviours display highly complex dynamics, making modelling difficult or impossible. Hence, the VSFP system is an ideal candidate to be used in conjunction with the PDBC method.

### Novelty and limitations

As described in the introduction, there are a range of data-driven algorithms for system understanding. Dynamic Mode Decomposition (DMD) [5] can be used to discovers physically meaningful modes and governing equations [3] from high dimensional time series datasets. Meanwhile, the work of Schmidt and Lipson [1] can distill free-form natural laws directly from time series data. PDBC is conceptually similar to these methods—in that it aids in understanding complex systems—but also fundamentally different in its application.

PDBC is designed to give global insight into systems whose behaviours change across their parameter space. The inteded usage is for systems with significantly different behaviours, such as those demonstrated in the VSPF system. In their current forms, the afformentioned alternatives are not well suited to this application. Rather, they would be effective in understanding the dynamics driving a particular behavioural mode. This is highly valuable, but does not provide the same global snapshot as PDBC. Indeed, the comparison is in some ways redundant as the two methods are in fact complimentary; PDBC provides the global picture, while alternative methods provide more specific insight of each behaviour.

More direct comparisons can be made with other approaches from the machine learning community. Support-vector machine (SVM) can be used to classify behaviours in real world systems via feature extraction. Gait anaylsis is one such example, with [56] using SVM for the autmated classification of gait in young versus elderly human subjects. The extracted features and decision boundaries are kin to PDQ’s in that they define a behavioural parameter space. However, the physical relevance of these features is hard to interpret. In this type of application, PDBC would perform the classifcation while also outputing physically interpreatble PDQ’s. This would also be the case for other machine learning classifiers such as neural networks.

Having said this, there are systems for which the current PDBC algorithm is inapplicable. Clearly, systems with no clear behavioural diversity are ruled out. More subtly, however, are systems with non-discrete behavioural modes. Here, there may be a clear range of behaviours separated by a continous transitonal zone, in which one behaviours blends into the next; period doubling, for example. Within this transitional zone behavoural classifcaiton is ambigous, making the data acqusition step of PDBC challenging. One approach may be to restrict sampling to areas within the parameter space with very clear behavours.

### Human bias

As previosly discussed, in PDBC the user must initially assign behaviours to experimental observations. In the VSFP system this was a relatively simple task, as the observed behaviour were clearly different from each other, allowing a completely unambigous classification. However, in general the users role in behavoural classifcaiton is siignificant. The user must decide what constitutes a behaviour, then apply this to the system observations. Hence, in the case of behavioural ambiguity, there may be no consensus among users regarding the total number of behaviours in the system.

As a short term solution, there are a few options. Firstly, to use a panel of observers to classify behaviours and reach consessus together. Alternatively, the PDBC algorithm can be run multiple times for each consensus. The solutions can be compared in terms of their solution landscape and physical significance. In the long term, however, the autmated interpretation of behaviours presents an interesting challenge. In the case of the VSFP system, motion capture systems could provide a wealth of trajectory data for such a system. The authors hope to implement this into PDBC in the future.

### Applications

The PDBC algorithm was designed with tha aim providing physical insightful behavoural classifcation for behavourally diverse systems. There are many applications in which this is desirable. Automated design optimisation, for example, often focusses on hard to model problems such as the real-world evolution of locomotion [12]. Behavourally diverse systems could multiple solutions to such problems. PDBC could be used in conjunction with quality diversity algorithms such as MAP-Elites [57, 58] to optimises such systems and provide a physically inshgtful snapshot of the solution landscape.

## Conclusion

For systems which do exhibit discrete behavioural modes, this approach opens up new avenues of analysis and understanding. However, further work is required to apply the method to systems with ambiguous or continuous behavioural phases. Additionally, further work is required in the choice of system parametrisation, output selection and behavioural interpretation. One of the main issues here is the human interpretation of system behaviours. Although relatively clear in the VSFP system, more complex system may exhibit a range of similar behaviours which are hard to distinguish between. Hence, there is scope to automate the process deciding what constitutes a discrete behavioural mode.

To fully realize the impact of this approach a more generalised method of approaching and achieving morphological range is required so it is not prescribed or influenced by initial human bias.

## Supporting information

S1 Video Video of four falling behaviours. (MP4)

S1 TableTable of $\bar{\dot{z}}$ and $\bar{\dot{\gamma }}$ for all experiments.(XLSX)

## References

[1] Schmidt M, Lipson H. Distilling free-form natural laws from experimental data. science. 2009;324(5923):81–85. 10.1126/science.1165893 19342586

[2] Bongard JC, Lipson H. Nonlinear system identification using coevolution of models and tests. IEEE Transactions on Evolutionary Computation. 2005;9(4):361–384. 10.1109/TEVC.2005.850293

[3] Brunton SL, Proctor JL, Kutz JN. Discovering governing equations from data by sparse identification of nonlinear dynamical systems. Proceedings of the National Academy of Sciences. 2016; p. 201517384. 10.1073/pnas.1517384113 PMC4839439 27035946

[4] Schmid Peter J. Dynamic mode decomposition of numerical and experimental data. Journal of fluid mechanics. 2010;656:5–28. 10.1017/S0022112010001217

[5] Kutz JN, Brunton SL, Brunton BW, Proctor JL. Dynamic mode decomposition: data-driven modeling of complex systems. vol. 149 SIAM; 2016.

[6] Gandomi A, Haider M. Beyond the hype: Big data concepts, methods, and analytics. International Journal of Information Management. 2015;35(2):137–144. 10.1016/j.ijinfomgt.2014.10.007

[7] Quiroz M, Kohn R, Villani M, Tran MN. Speeding up MCMC by efficient data subsampling. Journal of the American Statistical Association. 2018;(just-accepted):1–35. 10.1080/01621459.2018.144882730034060

[8] Yang Y, Ma Z, Nie F, Chang X, Hauptmann AG. Multi-class active learning by uncertainty sampling with diversity maximization. International Journal of Computer Vision. 2015;113(2):113–127. 10.1007/s11263-014-0781-x

[9] Strogatz SH. Nonlinear dynamics and chaos: with applications to physics, biology, chemistry, and engineering. CRC Press; 2018.

[10] Goza Andres and Colonius Tim. Modal decomposition of fluid–structure interaction with application to flag flapping. Journal of Fluids and Structures. 2018;81:728–737. 10.1016/j.jfluidstructs.2018.06.014

[11] Rosendo A, Von Atzigen M, Iida F. The trade-off between morphology and control in the co-optimized design of robots. PloS one. 2017;12(10):e0186107 10.1371/journal.pone.0186107 29023482 PMC5638323

[12] Brodbeck L, Hauser S, Iida F. Morphological evolution of physical robots through model-free phenotype development. PloS one. 2015;10(6):e0128444 10.1371/journal.pone.0128444 26091255 PMC4474803

[13] Nurzaman SG, Culha U, Brodbeck L, Wang L, Iida F. Active sensing system with in situ adjustable sensor morphology. PLoS One. 2013;8(12):e84090 10.1371/journal.pone.0084090 24416094 PMC3887119

[14] Kutz JN. Deep learning in fluid dynamics. Journal of Fluid Mechanics. 2017;814:1–4. 10.1017/jfm.2016.803

[15] Sano M, Tamai K. A universal transition to turbulence in channel flow. Nature Physics. 2016;12(3):249 10.1038/nphys3659

[16] Alexander R. Optimization and gaits in the locomotion of vertebrates. Physiological reviews. 1989;69(4):1199–1227. 10.1152/physrev.1989.69.4.1199 2678167

[17] Corucci Francesco and Cheney Nick and Giorgio-Serchi Francesco and Bongard Josh and Laschi Cecilia. Evolving soft locomotion in aquatic and terrestrial environments: effects of material properties and environmental transitions. Soft Robotics. 2018;5(4):475–495. 10.1089/soro.2017.0055 29985740

[18] Toner J, Tu Y, Ramaswamy S. Hydrodynamics and phases of flocks. Annals of Physics. 2005;318(1):170–244. 10.1016/j.aop.2005.04.011

[19] Maxwell JC. On a particular case of the descent of a heavy body in a resisting medium. The scientific papers of James Clerk Maxwell. 1854;9:115–118.

[20] Field SB, Klaus M, Moore MG, Nori F. Chaotic dynamics of falling disks. Nature. 1997;388(6639):252–254. 10.1038/40817

[21] Zhong H, Chen S, Lee C. Experimental study of freely falling thin disks: Transition from planar zigzag to spiral. Physics of Fluids. 2011;23(1). 10.1063/1.3541844

[22] Auguste F, Magnaudet J, Fabre D. Falling styles of disks. Journal of Fluid Mechanics. 2013;719:388–405. 10.1017/jfm.2012.602

[23] Lee C, Su Z, Zhong H, Chen S, Zhou M, Wu J. Experimental investigation of freely falling thin disks. Part 2. Transition of three-dimensional motion from zigzag to spiral. Journal of Fluid Mechanics. 2013;732:77–104. 10.1017/jfm.2013.390

[24] Zhong H, Lee C, Su Z, Chen S, Zhou M, Wu J. Experimental investigation of freely falling thin disks. Part 1. the flow structures and Reynolds number effects on the zigzag motion. Journal of Fluid Mechanics. 2013;716:228–250. 10.1017/jfm.2012.543

[25] Kanso E, Heisinger L, Newton P. Coins falling in water. Journal of Fluid Mechanics. 2014;742:243–253. 10.1017/jfm.2014.115

[26] Willmarth WW, Hawk NE, Harvey RL. Steady and Unsteady Motions and Wakes of Freely Falling Disks. Physics of Fluids. 1964;7(2):197 10.1063/1.1711133

[27] Vincent L, Shambaugh WS, Kanso E. Holes stabilize freely falling coins. Journal of Fluid Mechanics. 2016;801:250–259. 10.1017/jfm.2016.432

[28] Mahadevan L, Aref H, Jones SW. Comment on “behavior of a falling paper”; 1995. Available from: https://journals.aps.org/prl/pdf/10.1103/PhysRevLett.75.1420. 10.1103/PhysRevLett.75.1420 10060289

[29] Huang W, Liu H, Wang F, Wu J, Zhang HP. Experimetal study of a freely falling plate with an inhomogeneous mass distribution. Physical Review E—Statistical, Nonlinear, and Soft Matter Physics. 2013;88(5). 10.1103/PhysRevE.88.053008 24329352

[30] Chrust M, Bouchet G, Dûek J. Numerical simulation of the dynamics of freely falling discs. Physics of Fluids. 2013;25(4). 10.1063/1.4799179

[31] Pesavento U, Wang ZJ. Falling paper: Navier-Stokes solutions, model of fluid forces, and center of mass elevation. Physical Review Letters. 2004;93(14). 10.1103/PhysRevLett.93.144501 15524800

[32] Tanabe Y, Kaneko K. Behavior of a falling paper. Physical Review Letters. 1994;73(10):1372–1375. 10.1103/PhysRevLett.73.1372 10056776

[33] Belmonte A, Eisenberg H, Moses E. From Flutter to Tumble: Inertial Drag and Froude Similarity in Falling Paper. Physical Review Letters. 1998;81(2):345–348. 10.1103/PhysRevLett.81.345

[34] Andersen A, Pesavento U, Wang ZJ. Unsteady aerodynamics of fluttering and tumbling plates. Journal of Fluid Mechanics. 2005;541(-1):65–90. 10.1017/S002211200500594X

[35] Fernandes PC, Ern P, Risso F, Magnaudet J. On the zigzag dynamics of freely moving axisymmetric bodies. Physics of Fluids. 2005;17(9):1–4. 10.1063/1.2061609

[36] Mahadevan L, Ryu WS, Samuel ADT. Tumbling cards. Physics of Fluids. 1999;11(1):1–3. 10.1063/1.869919

[37] Kuznetsov SP. Plate falling in a fluid: Regular and chaotic dynamics of finite-dimensional models. Regular and Chaotic Dynamics. 2015;20(3):345–382. 10.1134/S1560354715030090

[38] Andersen A, Pesavento U, Wang ZJ. Analysis of transitions between fluttering, tumbling and steady descent of falling cards. Journal of Fluid Mechanics. 2005;541:91–104. 10.1017/S0022112005005847

[39] Skews BW. Autorotation of rectangular plates. Journal of Fluid Mechanics. 1990;217:33–40. 10.1017/S0022112090000611

[40] Jones MA, Shelley MJ. Falling cards. Journal of Fluid Mechanics. 2005;540:393–425. 10.1017/S0022112005005859

[41] Wang WB, Hu RF, Xu SJ, Wu ZN. Influence of aspect ratio on tumbling plates. Journal of Fluid Mechanics. 2013;733:650–679. 10.1017/jfm.2013.461

[42] Finn DL. Falling Paper and Flying Business Cards. SIAM News. 2007;40(4):4–6.

[43] Varshney K, Chang S, Wang ZJ. Unsteady aerodynamic forces and torques on falling parallelograms in coupled tumbling-helical motions. Physical Review E—Statistical, Nonlinear, and Soft Matter Physics. 2013;87(5). 10.1103/PhysRevE.87.053021 23767634

[44] Pesavento U, Wang ZJ. Falling Paper: Navier-Stokes Solutions, Model of Fluid Forces, and Center of Mass Elevation. Phys Rev Lett. 2004;93:144501 10.1103/PhysRevLett.93.144501 15524800

[45] Mittal R, Seshadri V, Udaykumar HS. Flutter, tumble and vortex induced autorotation. Theoretical and Computational Fluid Dynamics. 2004;17(3):165–170. 10.1007/s00162-003-0101-5

[46] Lauga E, Nadal F. Clustering instability of focused swimmers. EPL (Europhysics Letters). 2017;116(6):64004 10.1209/0295-5075/116/64004

[47] Goto Y, Tanaka H. Purely hydrodynamic ordering of rotating disks at a finite Reynolds number. Nature communications. 2015;6:5994 10.1038/ncomms6994 25629213

[48] Smith EH. Autorotating wings: An experimental investigation. Journal of Fluid Mechanics. 1971;50(3):513–534. 10.1017/S0022112071002738

[49] Iversen JD. Autorotating flat-plate wings: The effect of the moment of inertia, geometry and Reynolds number. Journal of Fluid Mechanics. 1979;92(2):327–348. 10.1017/S0022112079000641

[50] Norberg RA. Autorotation, self stability, and structure of single winged fruits and seeds (Samaras) with comparative remarks on animal flight. Biological Reviews of the Cambridge Philosophical Society. 1973;48(4):561–596.

[51] McCutchen CW. The Spinning Rotation of Ash and Tulip Tree Samaras. Science. 1977;197(4304):691–692. 10.1126/science.197.4304.691 17776273

[52] Lentink D, Dickson WB, Van Leeuwen JL, Dickinson MH. Leading-edge vortices elevate lift of autorotating plant seeds. Science. 2009;324(5933):1438–1440. 10.1126/science.1174196 19520959

[53] Gerhart PM, Gerhart AL, Hochstein JI. Munson, Young and Okiishi’s Fundamentals of Fluid Mechanics, Binder Ready Version. John Wiley & Sons; 2016.

[54] Hartigan JA, Wong MA. Algorithm AS 136: A k-means clustering algorithm. Journal of the Royal Statistical Society Series C (Applied Statistics). 1979;28(1):100–108.

[55] Rousseeuw PJ. Silhouettes: a graphical aid to the interpretation and validation of cluster analysis. Journal of computational and applied mathematics. 1987;20:53–65. 10.1016/0377-0427(87)90125-7

[56] Begg Rezaul K and Palaniswami Marimuthu and Owen Brendan. Support vector machines for automated gait classification IEEE transactions on Biomedical Engineering. 2005;828–838. 10.1109/TBME.2005.845241 15887532

[57] Mouret, Jean-Baptiste and Clune, Jeff Illuminating search spaces by mapping elites. arXiv preprint arXiv:1504.04909. 2015.

[58] Cully Antoine and Demiris Yiannis. Quality and diversity optimization: A unifying modular framework. IEEE Transactions on Evolutionary Computation. 2018;22(2):245–259. 10.1109/TEVC.2017.2704781

